# Last Words

**Published:** 1887-12

**Authors:** 


HALL'S
JOURNAL OF HEALTH

TRUTH DEMANDS NO SACRIFICE; ERROR CAN MAKE NONE.

VOL. 34. DECEMBER, 1887. No. 12.

### LAST WORDS.

The present issue of HALLS JOURNAL OF HEALTH, closes the series of 1887. At the request of a large number of our regular subscribers, who are accustomed to have their yearly numbers bound in a volume, we publish herewith a title page, and a complete table of contents, which in such instances, will be found of value.

We trust it will not be considered out of place, for us to remind our readers that the subscriptions of many of them, expire with this number. It would gratify the publisher, and greatly encourage him in his work to receive the prompt renewal of them.

We cannot of course, expect our numerous readers to accept as true, all the facts presented in the columns of the JOURNAL, however honestly entertained and emphatically asserted, but it is not unreasonable to ask of all dissenting minds, a candid and so far as possible, unprejudiced consideration of them.

He who contents himself with that only, which, squares with his preconceived notions of what is rational or possible, becomes almost without realizing it, narrow and one sided, to a degree of being incapable of exercising a fair and impartial judgment upon facts more or less unusual which belong wholly outside of the range, to which he is accustomed to confine his researches.

That the JOURNAL has been fortunate in having few such, even among its old time patrons, is made apparent by the continuance of their patronage, since it has become in small measure, the vehicle of the truths of a new dispensation. The number of those who have dropped out of the large mailing list carried by the JOURNAL, during the last two years, does not exceed six hundred, whilst its greatly increased and daily increasing subscriptions, give the most satisfactory evidence of its usefulness and general acceptance. That it is wedded to no particular system in medicine, morals or religion, but is eclectic in all, its course for the past twelve months, amply illustrates. The great object before us, is to present the truth side of whatever cause the JOURNAL espouses, without the fear or favor of any school or sect, moral secular or religious, and it is bound to continue in this course, so long as its present proprietor is permitted to give it direction.

The next issue of the JOURNAL commences its thirty fifth year, and the indications are that it will be one of unusual prosperity. The clouds are lifting.

